# Body surface area-based versus concentration-based intraperitoneal perioperative chemotherapy in a rat model of colorectal peritoneal surface malignancy: pharmacologic guidance towards standardization

**DOI:** 10.18632/oncotarget.26667

**Published:** 2019-02-15

**Authors:** Lieselotte Lemoine, Elsy Thijssen, Robert Carleer, Jirka Cops, Veerle Lemmens, Peter Van Eyken, Paul Sugarbaker, Kurt Van der Speeten

**Affiliations:** ^1^ Department of Medicine and Life Sciences, Hasselt University, Hasselt, Belgium; ^2^ Department of Surgical Oncology, Ziekenhuis Oost-Limburg, Genk, Belgium; ^3^ Department of Applied and Analytical Chemistry, Institute for Materials Research (IMO), Hasselt University, Diepenbeek, Belgium; ^4^ Department of Medicine and Life Sciences, Biomedical Research Institute, Rehabilitation Research Center, Hasselt University, Hasselt, Belgium; ^5^ Department of Medicine and Life Sciences, Dynamic Bioimaging Laboratory, Advanced Optical Microscopy Centre, Biomedical Research Institute (BIOMED), Hasselt University, Hasselt, Belgium; ^6^ Department of Pathology, Ziekenhuis Oost-Limburg, Genk, Belgium; ^7^ Center for Gastrointestinal Malignancies, MedStar Washington Hospital Center, Washington, DC, USA

**Keywords:** peritoneal surface malignancy, colorectal cancer, HIPEC, oxaliplatin, dosimetry

## Abstract

Worldwide, cytoreductive surgery (CRS) and hyperthermic intraperitoneal perioperative chemotherapy (HIPEC) are used in current clinical practice for colorectal peritoneal surface malignancy (PSM) treatment. Although, there is an acknowledged standardization regarding the CRS, we are still lacking a much-needed standardization amongst the various intraperitoneal (IP) chemotherapy protocols, including the HIPEC dosing regimen. We should rely on pharmacologic evidence building towards such a standardization. The current IP chemotherapy dosing regimens can be divided into body surface area (BSA)-based and concentration-based protocols. A preclinical animal study was designed to evaluate pharmacologic advantage (PA), efficacy and survival. WAG/Rij rats were IP injected with the rat colonic carcinoma cell line CC-531. Animals were randomized into three groups: CRS alone or CRS combined with oxaliplatin-based HIPEC (either BSA- or concentration-based). There was no difference in PA between the two groups (p=0.283). Platinum concentration in the tumor nodule was significantly higher in the concentration-based group (*p*<0.001). Median survival did not differ between the treatment groups (*p*<0.250). This preclinical study, in contrast to previous thinking, clearly demonstrates that the PA does not provide any information about the true efficacy of the drug and emphasizes the importance of the tumor nodule as pharmacologic endpoint.

## INTRODUCTION

Worldwide, cytoreductive surgery (CRS) and hyperthermic intraperitoneal perioperative chemotherapy (HIPEC) are used in current clinical practice for selected patients diagnosed with peritoneal surface malignancy (PSM) of colorectal origin [1, 2]. This combined treatment modality has resulted in significant survival benefit, with a median overall survival of 41.7 months (results presented at the ASCO annual meeting in Chicago) [3]. Clearly defined standardization of CRS, based on the work of Sugarbaker *et al.* [4, 5], has resulted in high-quality reproducible surgery performed at expert centers worldwide. In contrast, there is still a large variety of HIPEC treatment modalities used in current clinical practice. Methodological variations to be considered are: technique (open versus closed), normothermic versus hyperthermic chemotherapy, drug selection, drug dose, exposure time and dosing regimen [1, 6]. Conceptually, to standardize HIPEC, a randomized trial would be required with each variable as the only discriminating factor, but it is clear that multiple well-designed randomized controlled trials will not be conducted. Rather, we should rely on validated analytical assays and well-designed preclinical studies to build pharmacologic data towards improved and standardized HIPEC regimens. Therefore, an experimental study was performed to pharmacologically evaluate toxicity, efficacy and survival of body surface area (BSA)-based and concentration-based intraperitoneal (IP) chemotherapy in a rat model of colorectal PSM.

Most groups use a drug dose based on calculated BSA (mg/m^2^) in analogy to systemic chemotherapy regimens. These regimens take BSA as a measure for the effective peritoneal contact area; the peritoneal surface area in the Dedrick formula [7]. The Dedrick formula describes; rate of mass transfer = PA( C_Per_ – C_Bl_); where: PA = permeability area (PA = effective peritoneal contact area A x permeability P), C_Per_ = concentration in peritoneal cavity and, C_Bl_ = concentration in the blood [8]. Rubin *et al*. however, demonstrated there is an imperfect correlation between actual peritoneal surface area and calculated BSA [9, 10]. There may also be sex differences in peritoneal surface areas, which in turn affects absorption characteristics [11]. As a result, BSA-based IP chemotherapy will deliver a fixed dose (BSA-based) diluted in varying volumes of perfusate; i.e.; different concentrations depending on substantial differences in the body composition of patients and differences in the HIPEC technique (open versus closed abdomen). This implicates a high predictability of systemic chemotherapy levels and thus toxicity but a low predictability of peritoneal levels and thus tumor exposure to the drug. From the Dedrick formula we know that peritoneal concentration and not peritoneal dose is the driving diffusion force [7]. In that context, concentration-based chemotherapy offers a more predictable exposure of the tumor nodules to the IP chemotherapy and thus efficacy [12]. Unfortunately, the prize to be paid for a better prediction of the efficacy of the IP chemotherapy is a higher unpredictability of the systemic toxicity.

The aim of this manuscript is to pharmacologically evaluate toxicity, efficacy and survival of BSA-based and concentration-based IP chemotherapy in a rat model of colorectal PSM.

## RESULTS

### Oxaliplatin *in vitro* cytotoxicity

**Figure 1:**
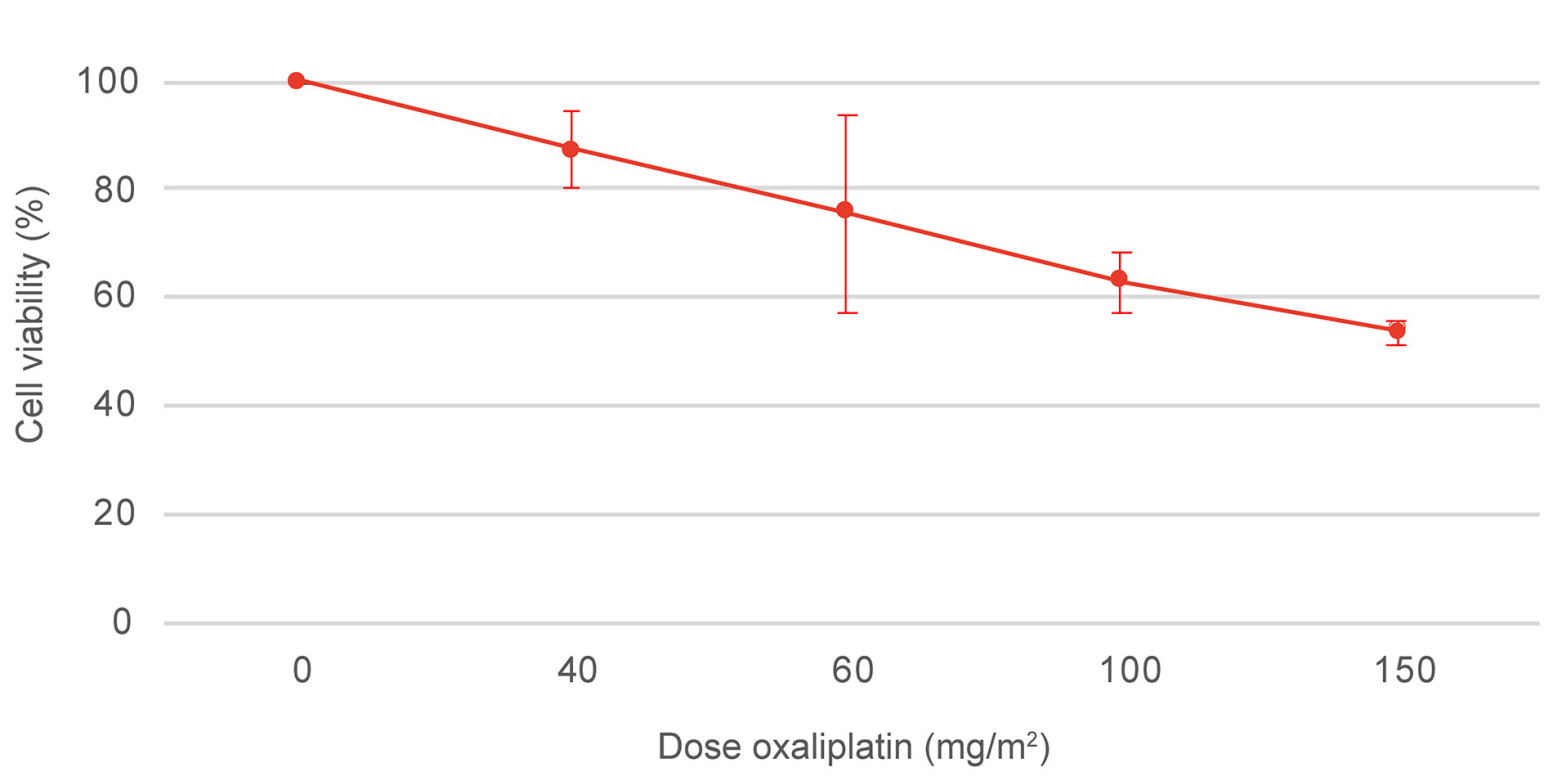
MTT assay of the CC-531 cell line after oxaliplatin treatment Cell viability was assessed after exposure to increasing concentrations of oxaliplatin, 0, 40, 60, 100 and 150 mg/m^2^ in 2 L/m^2^. Values are mean ± SD (n=3).

Viability of the CC-531 cell line after oxaliplatin treatment was evaluated *in vitro* by the 3-[4,5-dimethylthiazol-2-yl]-2,5-diphenyltetrazolium bromide (MTT) assay. Cell viability after exposure to increasing concentrations of oxaliplatin is presented in Figure 1. After treating the cells with the highest oxaliplatin dose, 75 μg/mL (150 mg/m^2^ in 2 L/m^2^), 53.6 ± 2.1% of the cells were still alive when compared to the control group.

### Maximum tolerated dose

**Figure 2:**
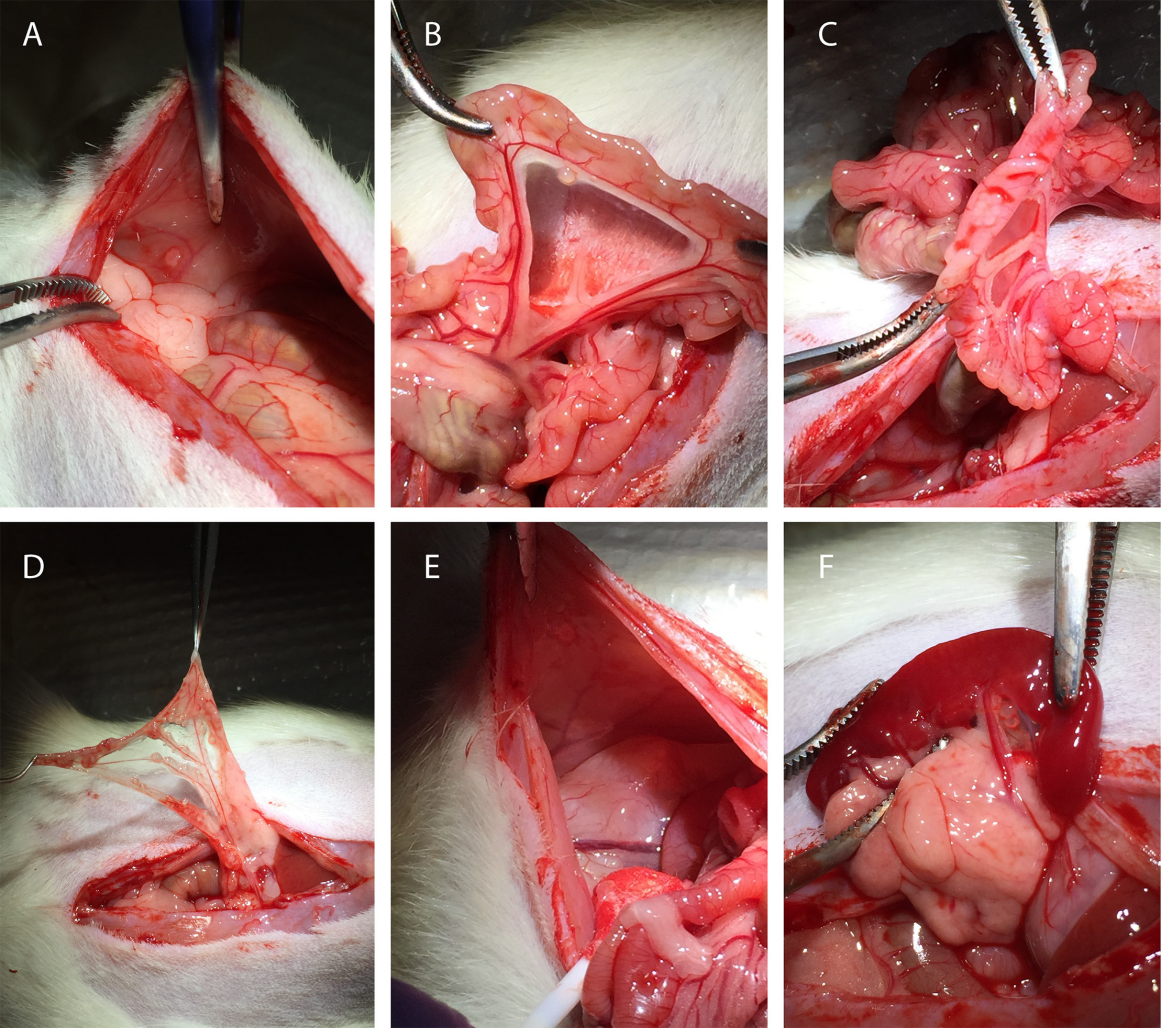
Macroscopic peritoneal tumor nodules found during laparotomy **(A)** injection site; **(B)** mesentery; **(C)** bowel surface; **(D)** greater omentum; **(E)** right abdominal fat pad; **(F)** spleen.

**Figure 3:**
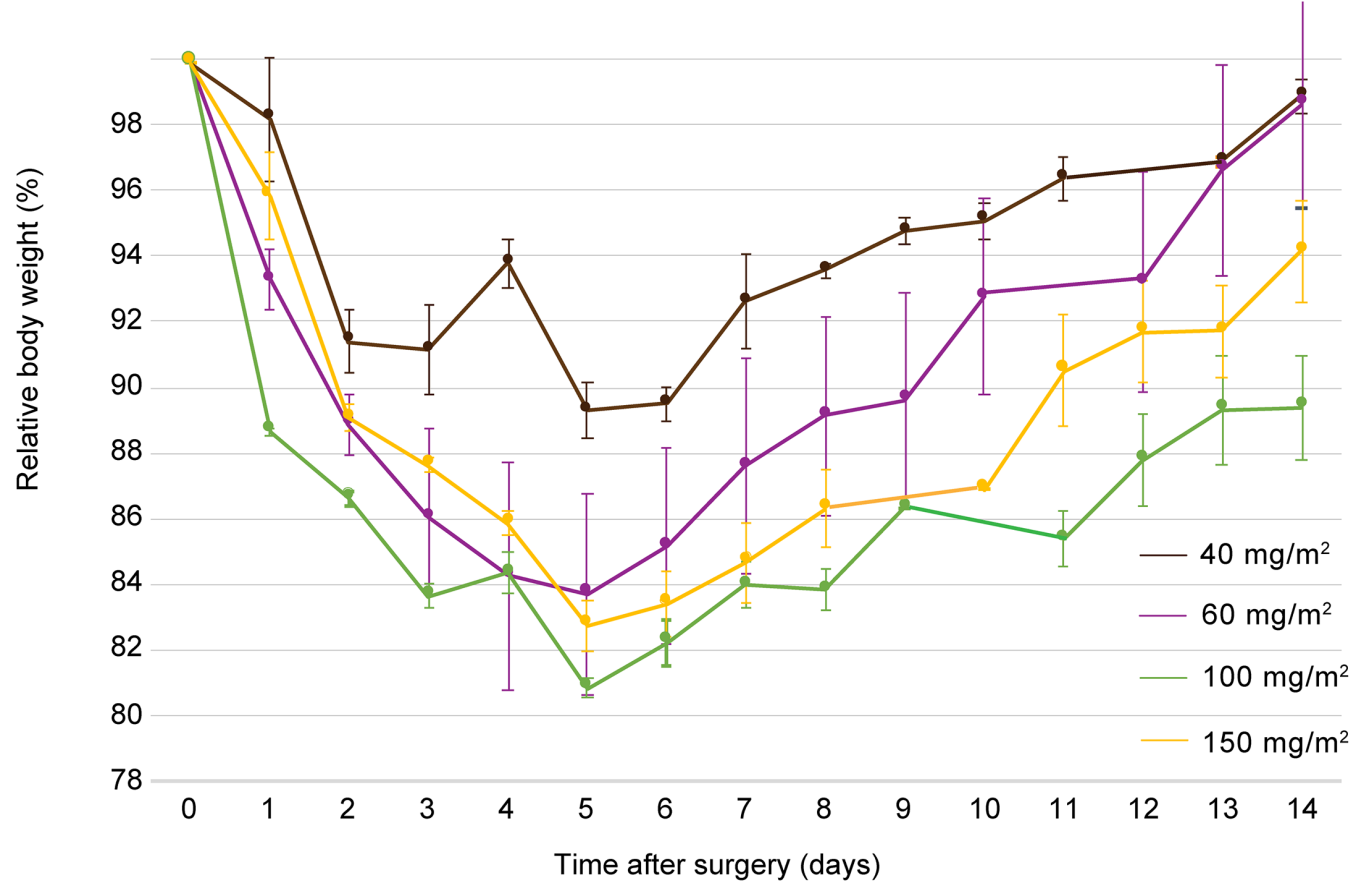
Evolution of mean body weight, relative to the weight at day of treatment, 14 days after surgery of rats treated with CRS and HIPEC to determine the maximum tolerated dose of oxaliplatin during HIPEC Rats were treated with 40 mg/m^2^ (brown), 60 mg/m^2^ (purple), 100 mg/m^2^ (green) and 150 mg/m^2^ (yellow) oxaliplatin. CRS, cytoreductive surger; HIPEC, hyperthermic intraperitoneal perioperative chemotherapy. Values are mean ± SE.

To determine *in vivo* toxicity of the CC-531 cell line, rats were treated with CRS and HIPEC with increasing doses of oxaliplatin (40 – 150 mg/m^2^ in 2 L/m^2^). At laparotomy, all animals had macroscopic tumor deposits. The injection site, greater omentum, liver hilum, perisplenic area and mesentery were identified as the most affected sites (Figure 2). Overall median modified peritoneal cancer index (PCI) before CRS was similar in all groups (*p* = 0.089): 40 mg/m^2^: 13.0 (11.5 – 13.0); 60 mg/m^2^: 5.0 (5.0 – 6.5); 100 mg/m^2^: 6.0 (6.0 – 8.5); 150 mg/m^2^: 7.0 (6.5 – 7.0). R2a resection was achieved in all animals, leaving residual tumor deposits smaller than 2.5 mm on the bowel surface. Intra-abdominal temperature at the outflow drain and rectal temperature were constant and similar in both subgroups, with a median temperature of 40.3°C (39.7 – 40.7) (*p* = 0.224) and 36.3°C (35.6 – 37.3) (*p* = 0.862), respectively. Figure 3 demonstrates the evolution of mean body weight in the four subgroups, 14 days postoperatively. The lowest mean body weight was recorded on the 5th postoperative day: reduction of 10.6 ± 0.8% (40 mg/m^2^), 16.2 ± 3.1% (60 mg/m^2^), 19.0 ± 0.8% (100 mg/m^2^) and 17.1 ± 0.3% (150 mg/m^2^). All animals generally gained weight from the 6th postoperative day onwards. Besides weight loss, commonly observed side effects were reduced activity and less grooming. All animals survived the 14-day period and were euthanized. Autopsy was performed in all but two rats, treated with CRS and 40 mg/m^2^ oxaliplatin-based HIPEC. PCI score at autopsy did not differ between the groups (*p* = 0.141): 7.0 (40 mg/m^2^), 5.0 (4.0 – 6.0) (60 mg/m^2^), 5.0 (3.5 – 5) (100 mg/m^2^) and 1.0 (0.5 – 2.5) (150 mg/m^2^). Maximum tolerated dose (MTD) was defined at 150 mg/m^2^ oxaliplatin in 2 L/m^2^ 0.9% (w/v) NaCl carrier solution.

### Body surface area-based versus concentration-based HIPEC

#### Cytoreductive surgery

At laparotomy, all animals had macroscopic tumor deposits with the greater omentum, liver hilum, perisplenic area, mesentery, bowel surface and gonadal fat pads identified as the most affected sites (Table 1). Overall mean modified PCI before CRS was significantly different between the HIPEC-CONC group, the CRS group (*p* < 0.001) and the HIPEC-BSA group (*p* = 0.029): CRS group: 8.1 ± 2.4; HIPEC-BSA: 9.7 ± 2.3; HIPEC-CONC: 11.5 ± 2.0. Completeness of resection, denoted by the R-score, did not differ between the groups (*p* = 0.543). As no bowel resections were performed and no anastomosis were made, a R1 resection could not be achieved (Table 1). R2a resection was accomplished in 45 rats and a R2b resection was accomplished in 18 rats. Median duration of surgery was 39 (35 – 43) minutes.

**Table 1:** Modified peritoneal cancer index score before cytoreductive surgery

Subgroup	CRS (n=21)	HIPEC-BSA (n=21)	HIPEC-CONC (n=21)
Tumour score per site^a^			
Subcutaneous	0 (0-3)	0 (0-3)	0 (0-0)
Injection site	0 (0-3)	1 (0-1)	1 (0-1)
Greater omentum	2 (0-2)	1 (0-1)	2 (1-2)
Liver hilum	2 (0-2)	1 (0-3)	2 (1-3)
Liver	0 (0-0)	0 (0-0)	0 (0-0)
Perisplenic	1 (1-2)	1 (0-2)	1 (1-3)
Mesentery	1 (0-1)	1 (0-3)	1 (0-3)
Bowel surface	1 (1-1)	1 (0-1)	1 (1-3)
Abdominal fat pads	0 (0-0)	3 (0-3)	0 (0-3)
Gonadal fat pads	1 (0-3)	1 (1-3)	2 (1-3)
Diaphragm	0 (0-1)	0 (0-0)	0 (0-2)
Parietal peritoneum	0 (0-1)	0 (0-3)	0 (0-1)
Mean PCI^b^	8.1 (2.4)	9.7 (2.3)	11.5 (2.0)
Completeness of resection (n)			
R1	0	0	0
R2a	14	14	17
R2b	7	7	4

CRS, cytoreductive surgery; HIPEC, hyperthermic intraperitoneal perioperative chemotherapy; BSA, body surface area; PCI, peritoneal cancer index. Values are ^a^median (range), ^b^mean (SD); 0, no macroscopic tumor; 1, limited tumor growth (1–2 mm Ø); 2, moderate tumor growth (2–4 mm Ø); 3, abundant presence of tumor nodules (>4 mm Ø); the sum of scores from all sites represent the overall modified PCI. Completeness of resection after CRS; R1, absence of residual tumor; R2a, residual tumor of 2.5 mm or less; R2b, residual tumor larger than 2.5 mm.

#### Hyperthermic intraperitoneal perioperative chemotherapy

**Figure 4:**
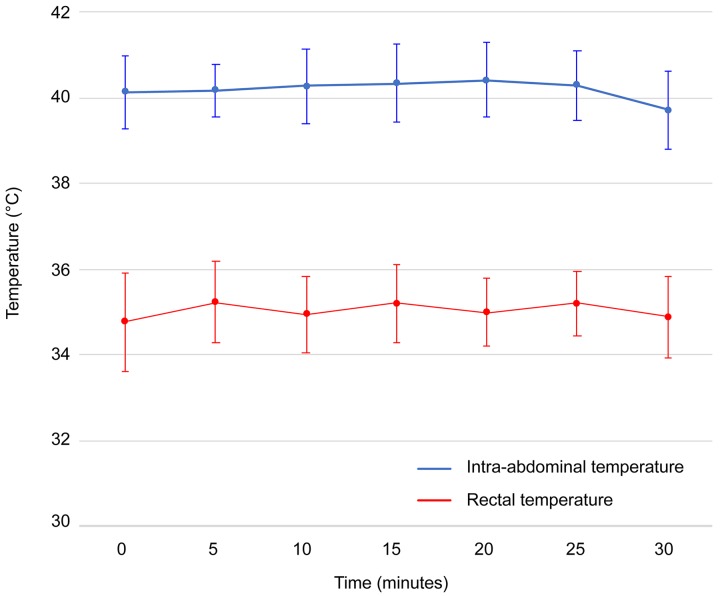
Intra-abdominal and rectal temperatures monitored during hyperthermic intraperitoneal perioperative chemotherapy Intra-abdominal temperatures (°C) are depicted in blue and rectal temperatures (°C) are depicted in red. Values are mean ± SD (n=42).

During HIPEC, intra-abdominal temperature at the outflow drain and rectal temperature were constant and similar in all subgroups, with a mean intra-abdominal temperature of 40.2 ± 0.7 °C (*p* = 0.276) and a mean rectal temperature of 35.9 ± 0.7°C (*p* = 0.434) (Figure 4). Mean duration of anesthesia was 114.1 ± 11.4 minutes.

#### Pharmacology of oxaliplatin during HIPEC

**Figure 5:**
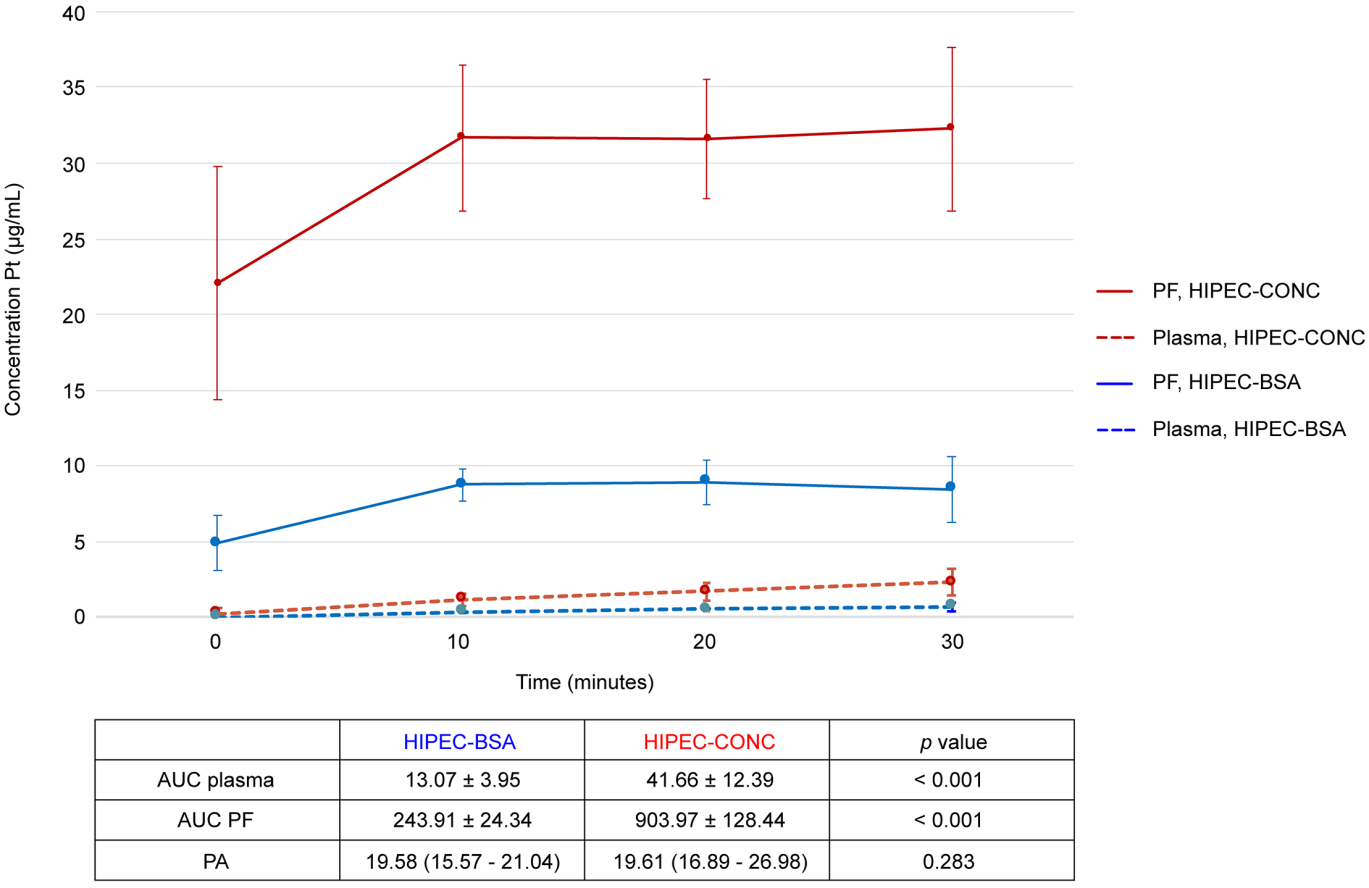
Pharmacokinetic graph of oxaliplatin-derived Pt in peritoneal fluid and plasma during the 30-minute HIPEC Pt concentrations in peritoneal fluid (PF, full line) and plasma (dotted line) of rats treated with concentration-based HIPEC are depicted in red. Pt concentrations in PF (full line) and plasma (dotted line) of rats treated with BSA-based HIPEC are depicted in blue. Pt, platinum; PF, peritoneal fluid; HIPEC, hyperthermic intraperitoneal perioperative chemotherapy; AUC, area-under-the curve; BSA, body surface area; PA, pharmacologic advantage. Values are mean ± SD (n=21 per group).

Oxaliplatin-derived platinum (Pt) concentrations in plasma and peritoneal fluid, of rats treated with either concentration- or BSA-based HIPEC, are depicted in Figure 5. Area-under-the curve (AUC) of the plasma and peritoneal fluid compartment, reflecting toxicity and efficacy of the treatment, was significantly different between the treatment groups (*p* < 0.001). The ratio of AUC peritoneal fluid over AUC plasma, the pharmacologic advantage (PA), was similar in both groups (*p* = 0.283) with a median PA of 19.60 (16.02 – 25.82). Pt concentration in the tumor nodule was significantly higher in the HIPEC-CONC group; 20.44 ± 9.10 ng/mg, as compared to the HIPEC-BSA group; 4.74 ± 2.49 ng/mg (*p* < 0.001).

#### Apoptosis measurements

**Figure 6:**
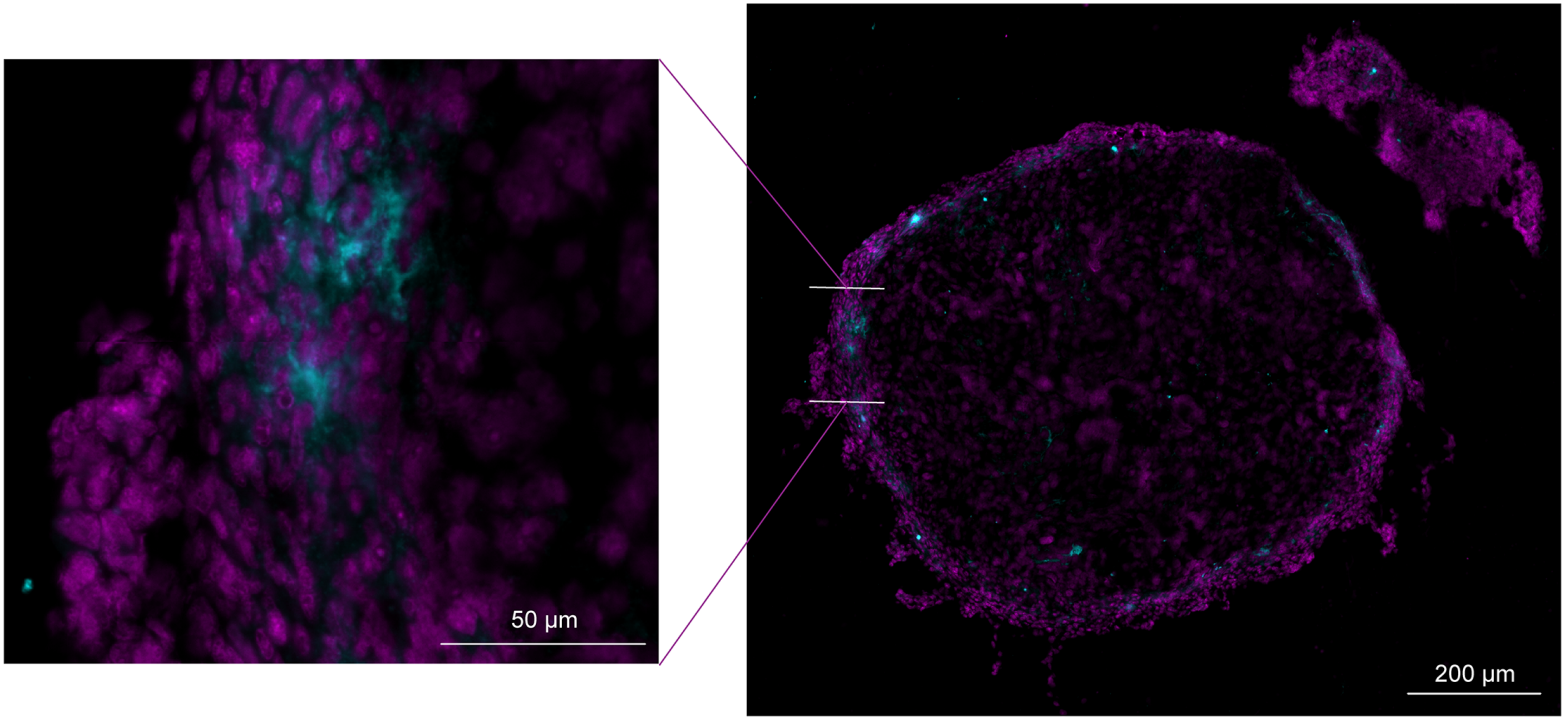
Representative image of immunohistochemistry staining for activated caspase-3 Cross section (10 μm thick) of inner layer of a greater omentum tumor nodule of a rat treated with BSA-based HIPEC. DAPI staining (magenta) and activated caspase-3 staining (cyan). BSA, body surface area; HIPEC, hyperthermic intraperitoneal perioperative chemotherapy.

**Figure 7:**
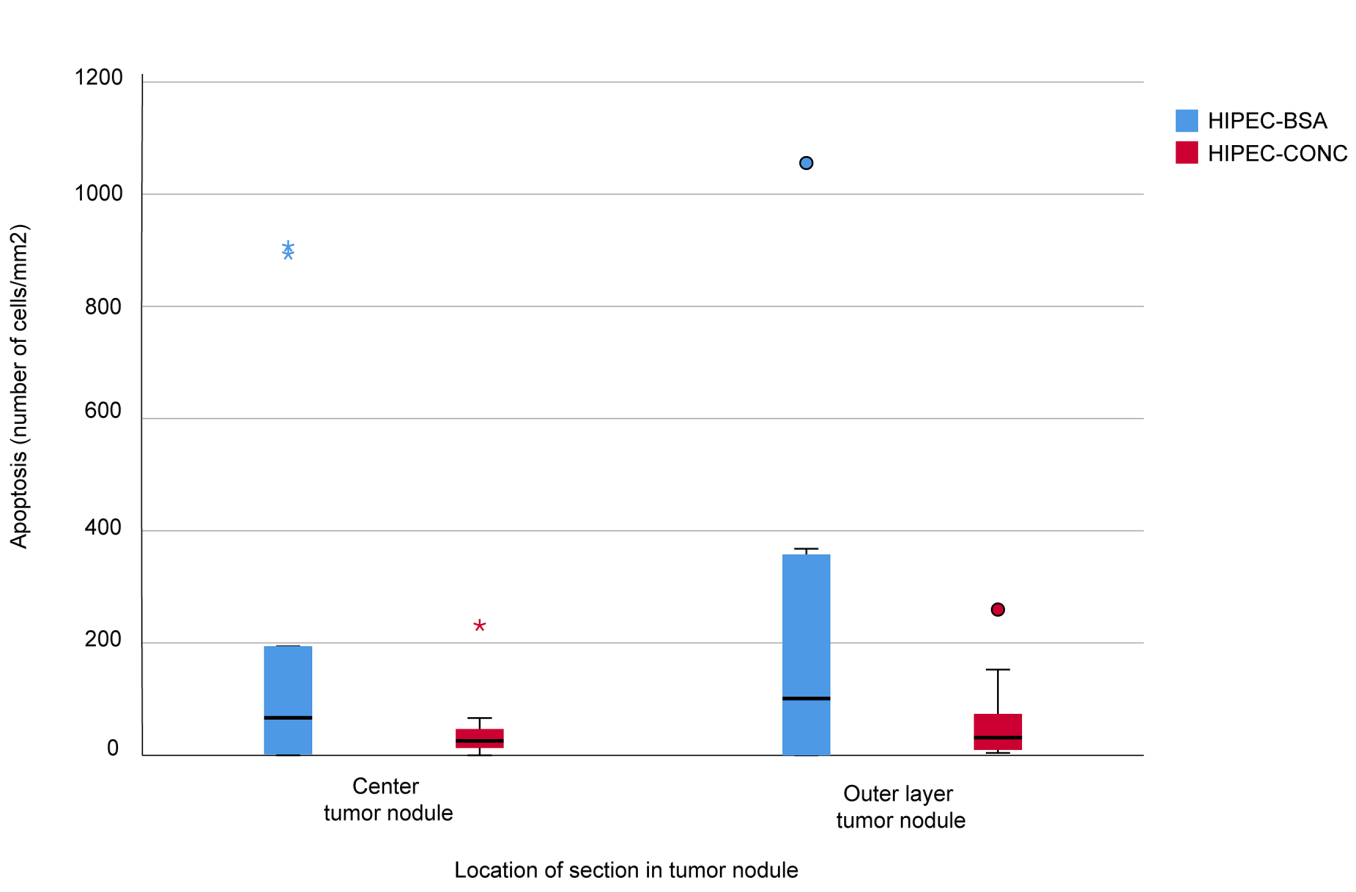
Apoptosis measurements Apoptosis in the center and outer layer of the tumor nodule was evaluated in rats treated with concentration-based HIPEC (HIPEC-CONC, n=10, red) and BSA-based HIPEC (HIPEC-BSA, n=10, blue) by means of immunohistochemistry for activated caspase-3. Median number of cells/mm^2^ was 25.56 (13.16 – 46.5) in the center and 31.70 (9.47 – 73.73) in the outer layer of the tumor nodule in the HIPEC-CONC group. In the HIPEC-BSA group, median number of cells/mm^2^ was 66.84 (1.67 – 194.29) in the center of the tumor nodule and 131.06 (10.67 – 357.62) in the outer layer of the nodule.

Apoptosis in the center and outer layer of the tumor nodule was evaluated in the HIPEC-CONC (n=10) and the HIPEC-BSA (n=10) group by means of immunohistochemistry (IHC) staining for activated caspase-3 (Figure 6). Median number of cells/mm^2^ was 25.56 (13.16 – 46.5) in the center and 31.70 (9.47 – 73.73) in the outer layer of the tumor nodule in the HIPEC-CONC group (Figure 7). In the HIPEC-BSA group, median number of cells/mm^2^ was 66.84 (1.67 – 194.29) in the center of the tumor nodule and 131.06 (10.67 – 357.62) in the outer layer of the nodule (Figure 7). There was no significant difference in amount of apoptosis, both in the center (*p* = 0.279) and outer layer (*p* = 0.388) of the tumor nodule, between the HIPEC treatment groups. Furthermore, within each treatment group, there was no difference between amount of apoptosis in the center and outer layer of the nodule, nor could a correlation between Pt concentration in the nodule at the end of HIPEC and amount of apoptosis be found.

#### Survival

**Figure 8:**
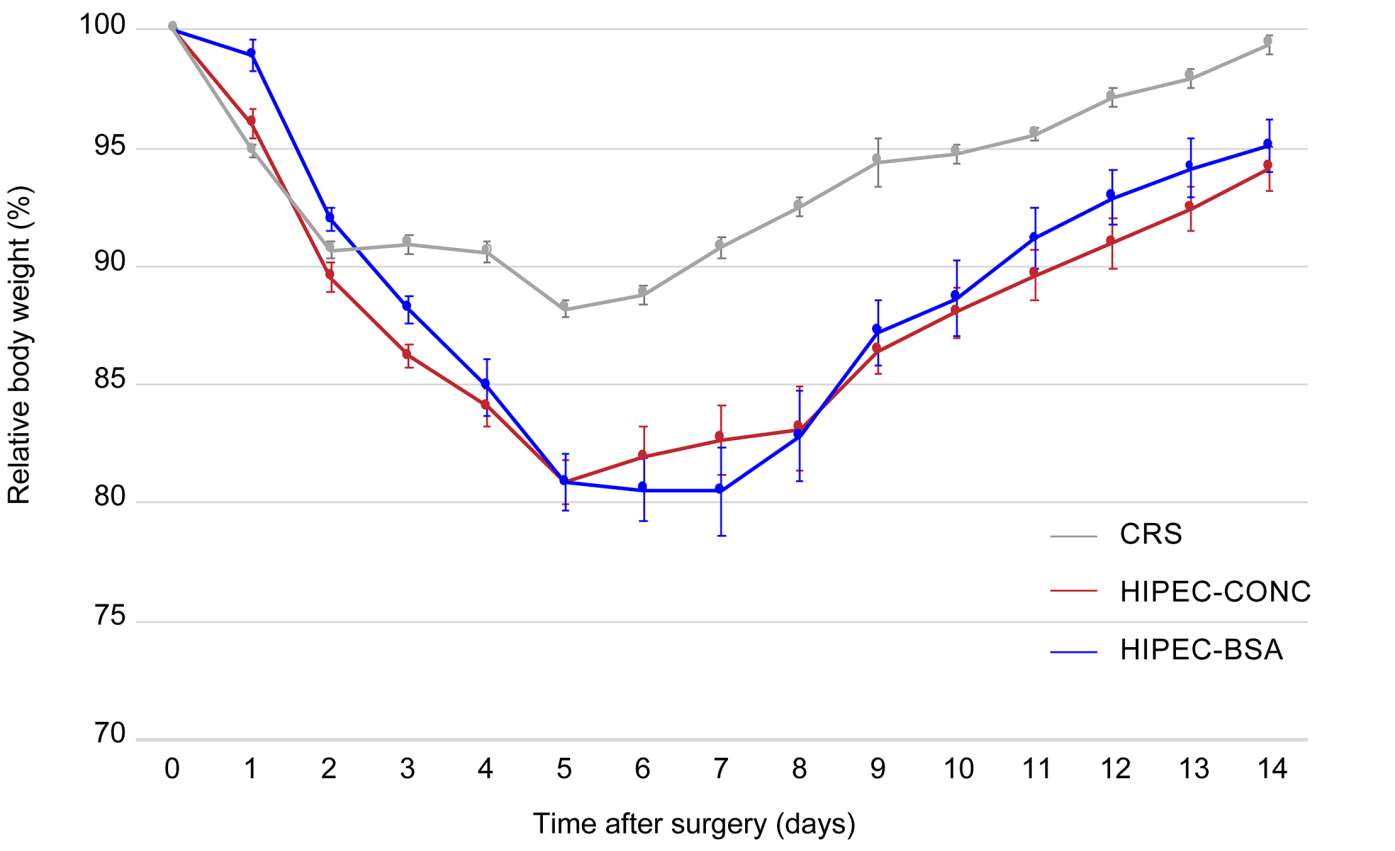
Evolution of mean body weight, relative to the weight at day of treatment, 14 days after surgery Relative body weight of rats treated with either CRS alone is depicted in grey, treated with CRS and HIPEC concentration-based (HIPEC-CONC) depicted in red and treated with CRS and HIPEC BSA-based (HIPEC-BSA) depicted in blue. CRS, cytoreductive surgery; HIPEC, hyperthermic intraperitoneal perioperative chemotherapy; BSA, body surface area. Values are mean ± SE.

The lowest mean body weight was recorded on the 5th (CRS and HIPEC-CONC) or 7th (HIPEC-BSA) postoperative day (Figure 8). Weight reduction was significantly lower in the CRS group, 11.8 ± 0.4% when compared to the HIPEC-CONC group, 19.1 ± 0.9% (*p* < 0.001) and the HIPEC-BSA group, 19.5 ± 1.9% (*p* = 0.003). Besides weight loss, commonly observed side effects were reduced activity and less grooming.

**Figure 9:**
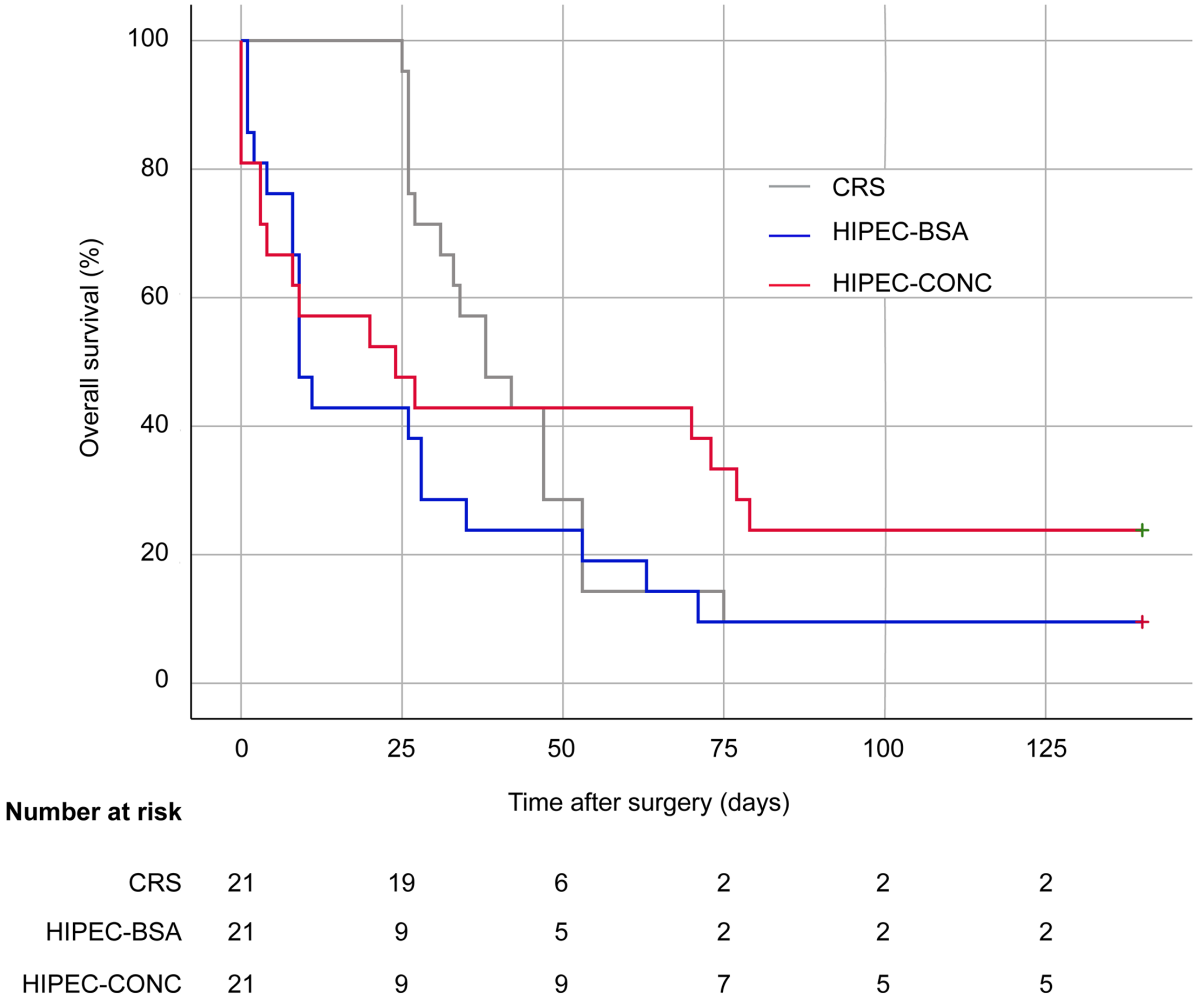
Kaplan-Meir curves Survival curves are presented of rats treated with CRS only (grey), CRS combined with HIPEC, BSA-based (blue) and CRS combined with HIPEC, concentration-based (red). CRS, cytoreductive surgery; HIPEC, hyperthermic intraperitoneal perioperative chemotherapy; BSA, body surface area.

Survival curves are depicted in Figure 9. Median survival was 38 (95% confidence interval (c.i.) 26 to 50) days in the CRS group, 9 (95% c.i. 6 to 12) days in the HIPEC-BSA group, 24 (95% c.i. 0 to 51) days in the HIPEC-CONC group; and did not differ between the groups (*p* = 0.250). After 140 days, 9 rats were still alive: 2 in CRS group, 2 in HIPEC-BSA group and 5 in HIPEC-CONC group. At autopsy, 4 of these 9 rats presented only with a very small nodule on the bowel surface: 1 in CRS group, 1 in HIPEC-BSA group, and 2 in HIPEC-CONC group. All rats in the CRS group survived the surgery, whereas 4 rats in the HIPEC-CONC group did not wake up from the anesthesia. Other non-tumor-related causes of death, within 2 days after CRS and HIPEC, were organ failure and bowel obstruction.

PCI score at autopsy was significantly lower in rats treated with CRS and HIPEC as compared to rats treated with CRS alone (*p* < 0.001) (Table 2). Within each treatment group, PCI was significantly lower at autopsy as compared to day of treatment when rats received CRS and HIPEC-CONC (*p* < 0.001) and CRS and HIPEC-BSA (*p* = 0.002). In contrast, when rats were treated with CRS alone, PCI at autopsy was significantly higher as compared to the PCI score at day of treatment (*p* < 0.001). In the latter group, significantly more volume of ascites was drained at autopsy (*p* < 0.001), and hemorrhagic ascites was therefore the most common reason for reaching the humane endpoint is this treatment group. Other reasons for ending the experiment were significant weight loss, diarrhea and palpable tumor nodules in the abdomen or subcutaneously. Using Cox survival regression analysis, no confounding factors influencing survival could be identified. Variables considered were modified PCI, R-score, AUC plasma, AUC peritoneal fluid, PA and Pt concentration in the tumor nodule at the end of HIPEC.

**Table 2:** Modified peritoneal cancer index score at autopsy

Subgroup	CRS (n=17)	HIPEC-BSA (n=21)	HIPEC-CONC (n=19)
Tumour score per site^a^			
Subcutaneous	3 (0-3)	0 (0-3)	0 (0-3)
Injection site	2 (0-3)	1 (0-3)	1 (0-3)
Greater omentum	0 (0-0)	0 (0-0)	0 (0-0)
Liver hilum	3 (0-3)	0 (0-3)	0 (0-3)
Liver	1 (0-3)	0 (0-0)	0 (0-1)
Perisplenic	0 (0-3)	0 (0-0)	0 (0-0)
Mesentery	2.5 (0-3)	0 (0-3)	0 (0-3)
Bowel surface	1 (1-3)	1 (1-3)	1 (0-3)
Abdominal fat pads	3 (0-3)	1 (0-3)	0 (0-3)
Gonadal fat pads	3 (0-3)	0 (0-3)	0 (0-3)
Diaphragm	3 (0-3)	0 (0-3)	0 (0-3)
Parietal peritoneum	1 (0-3)	0 (0-1)	0 (0-3)
Mean PCI^a^	19 (11-22)	3 (3-7)	3 (2-5)
Volume of ascites (mL)^a^	55 (0-65)	0 (0-0)	0 (0-3)

CRS, cytoreductive surgery; HIPEC, hyperthermic intraperitoneal perioperative chemotherapy; BSA, body surface area; PCI, peritoneal cancer index. Values are ^a^median (range); 0, no macroscopic tumor; 1, limited tumor growth (1–2 mm Ø); 2, moderate tumor growth (2–4 mm Ø); 3, abundant presence of tumor nodules (>4 mm Ø); the sum of scores from all sites represent the overall modified PCI.

## DISCUSSION

This is the first preclinical animal study designed to build pharmacologic data towards an improved and standardized HIPEC procedure, focusing on the dosimetry of IP chemotherapy. Using validated assays, we report that there is no difference in PA, defined as the ratio of AUC peritoneal fluid over AUC plasma, between BSA-based and concentration-based HIPEC. However, proof of principle is provided, that a higher IP concentration of the chemotherapeutic drug in concentration-based HIPEC results in a higher drug concentration in the tumor nodule at the end of HIPEC.

The Wag/Rij rat injected with the syngeneic CC-531 cell line is a widely used, validated and reproducible animal model of colorectal PSM, which resembles the clinical situation [13]. In the past, this preclinical model has frequently been used in combination with IP mitomycin C, the alternative HIPEC drug in the treatment of colorectal PSM [14–17]. Several research groups used the CC-531 cell line in combination with oxaliplatin [18–22]. However, as they did not include a cell viability assay to investigate sensitivity of the cell line for oxaliplatin, we performed a MTT assay and report the CC-531 cell line to be sensitive for oxaliplatin.

The syngeneic rat model of colorectal PSM enables one to quantify the extent of disease using the modified PCI score, perform surgery and HIPEC procedures, both open and closed [14, 23]. To create a standardized experimental model, we decided to surgically remove the greater omentum, spleen and gonadal fat pads by default. Additionally, macroscopic tumor deposits on the abdominal fat pads and other locations were removed or cauterized when possible, but no bowel resections or anastomoses were made. In our study design, it was therefore not feasible to achieve a R1 score, a complete macroscopic resection. However, tumor nodules left on the bowel surface were 1 to 2 mm in size, which is within penetration depth of oxaliplatin [24]. HIPEC in the animal model was performed by the open technique with a perfusion time of 30 minutes which mimics the current clinical setting in our institute. A disadvantage of using the open HIPEC technique in this animal model when compared to the closed technique, is that the target temperature of 41.5°C could not be reached without increasing the overall body temperature of the rats. Therefore, in this setting, HIPEC was performed at a median temperature of 40.3°C.

Initially, Hribaschek and colleagues reported the use of an IP oxaliplatin dose of 80 mg/m^2^ (BSA-based), but due to high mortality rates in the animals, they reduced the dose to 60 mg/m^2^. Grémonprez *et al.* determined 150 mg/m^2^ oxaliplatin in 2 L/m^2^ carrier solution (concentration-based) to be the MTD in a mouse model of colorectal PSM [25]. To determine MTD of oxaliplatin in our animal model, we used doses of oxaliplatin ranging from 40 to 150 mg/m^2^ in 2 L/m^2^, the latter being approximately 1/3 of the clinical high dose of 460 mg/m^2^ [26]. Figure 3 presents that rats treated with 100 mg/m^2^ lose more weight in the 14-day postoperative period when compared to rats treated with 150 mg/m^2^ oxaliplatin, although not significant. However, we defined 150 mg/m^2^ in 2 L/m^2^ as the MTD for future experiments because the PCI score at autopsy was the lowest in this treatment group. Furthermore, no blood sampling was performed during the MTD experiments and we expected this to further increase the weight reduction after CRS and HIPEC.

To pharmacologically evaluate the two dosing regimens that are used as standard of care in current clinical practice, we designed an animal study where rats were treated with CRS alone or CRS combined with HIPEC either BSA-based or concentration-based. The modified PCI score at day of treatment was significantly higher in the HIPEC-CONC group, but after surgery completeness of resection was similar in all treatment groups. In the HIPEC-CONC group, as every rat was treated with the same oxaliplatin concentration, we did not expect the large SD (22.08 ± 7.65 μg/mL) of the Pt concentration in the peritoneal fluid at the start of HIPEC (Figure 5). This Pt concentration variability suggests that 2 minutes homogenization is not sufficient. The Pt concentration measured in the peritoneal fluid sample taken at the start of the 30-minute HIPEC clock was only 63.65 ± 17.12% of the theoretical Pt concentration administered in the HIPEC reservoir. One could argue to prolong the homogenization period, but this will lead to loss of information in the plasma compartment as Pt levels, found at the beginning of HIPEC, are far above the limit of quantification of the ICP-MS method. Moreover, a longer homogenization period will increase systemic hyperthermia. Another possible solution, to overcome the need for homogenization of the chemotherapy solution, is to already dilute oxaliplatin in the carrier solution in a large reservoir before HIPEC treatment. However, 0.9% (w/v) NaCl was used in our experimental design as carrier solution and it is known that oxaliplatin undergoes degradation in chloride-containing solutions [27]. This degradation is nonetheless limited to approximately 10% after 30 minutes and degradation is believed to promote the formation of its active cytotoxic drug form. Pre-mixing of the chemotherapy solution will also not allow to reach stable perfusion conditions, such as stable hyperthermia, before starting chemotherapy treatment. Pharmacokinetics of IP drug delivery can be described by a simple 2-compartment model [28]. This model comprises a systemic compartment (characterized by a drug concentration and distribution volume) and a peritoneal compartment (characterized by a perfusate concentration and volume). As the peritoneal compartment is the biggest compartment, a possible recommendation would be to administer the drug directly in the peritoneal cavity to facilitate homogenization of the chemotherapy solution. This recommendation overcomes the previously mentioned drawbacks of prolonging the homogenization period, i.e. loss of information in the systemic compartment and risk of systemic hyperthermia. These findings might also have a profound implication on current clinical practice.

The ideal drug for IP drug delivery has a high peritoneal tissue concentration (high AUC of the peritoneal fluid compartment) and high penetration in the tumor nodule. This should occur in conjunction with slow diffusion of the chemotherapy solution through the peritoneal membrane and deep in the subperitoneal space, resulting in low systemic exposure (low AUC of the plasma compartment) [6]. In this setting, the AUC of the peritoneal fluid compartment reflects efficacy of the drug and the AUC of the systemic compartment reflects toxicity. This explains why the PA has been used as a parameter to assess the potential of a given drug to be used during HIPEC [28]. Systemic and peritoneal fluid Pt levels were significantly different between the HIPEC-BSA and HIPEC-CONC treatment groups. Nevertheless, concerning the primary endpoint of our preclinical study, this did not result in a significant difference in PA between the HIPEC treatment groups. However, at the same time we found higher levels in the tumor nodule in the HIPEC-CONC group. These data clearly demonstrate that the PA does not provide any information about the amount of chemotherapy reaching the tumor nodule in the peritoneal cavity. We provide proof of principle that a higher concentration of the chemotherapeutic drug in the peritoneal fluid (HIPEC-CONC), results in a higher concentration of the drug in the tumor nodule at the end of the 30-minute HIPEC procedure. Therefore, the tumor nodule should be considered the pharmacologic endpoint rather than the PA of a drug.

The next logical step was to investigate if a higher drug concentration in the tumor nodule also results in a higher amount of apoptosis. In this setting, apoptosis was considered a measure to comprehend all pharmacodynamic effects. Recent insights into the tumor biology suggest that pharmacodynamic parameters (vascularity, IFP, extracellular matrix properties, etc.) are at least as important in tumor response to chemotherapy as the pharmacokinetics mentioned above [29]. As a pilot study, we assessed the amount of cell death in the center and outer layer of 10 tumor nodules in each of the HIPEC treatment groups by means of IHC staining for activated caspase-3. Statistical analysis did not demonstrate a significant difference in amount of apoptosis in both layers of the tumor nodule treated with either BSA-based or concentration-based HIPEC. Although no difference in the amount of apoptotic cells could be detected, the range of apoptosis is smaller in the HIPEC-CONC group. This is as expected as every tumor nodule in this treatment group received a fixed concentration of oxaliplatin (75 mg/L, i.e. 150 mg/m^2^ in 2 L/m^2^). Although not confirmed by statistical analysis, there is a trend towards more apoptotic cells per mm^2^ in the outer layer when compared to in the center of the tumor nodule. The clinical implications of this apoptotic pilot study are very limited but nevertheless very important in that it is hypothesis generating. As tumor nodules were sampled at the end of the 30-minute HIPEC procedure, we should consider that at that point in time an effect of the drug cannot be detected. A limitation of this pilot study is that, due to practical reasons, it was not possible to sample tumor nodules at later time points after HIPEC, to evaluate apoptosis. Another limitation is that no sound conclusion can be made if the demonstrated apoptosis is due to the CRS or to the HIPEC part of the combined therapy; as no tumor nodules for apoptosis quantification were sampled in the CRS-only group.

In contrast to a previous preclinical study conducted by Klaver *et al.* [30], we could not demonstrate a median survival benefit for the HIPEC treatment groups when compared to the CRS group. A possible explanation could be the choice of drug, as Klaver and colleagues used mitomycin C as the chemotherapeutic agent in their HIPEC procedure. In our study, all rats in the CRS group survived the surgery, whereas 4 rats in the HIPEC-CONC group did not wake up from anesthesia and others in the HIPEC treatment groups died from organ failure or bowel obstruction. This can be explained by the fact that in the CRS group, the duration of anesthesia was much shorter as compared to the HIPEC groups and no sampling was performed. Furthermore, the mean weight during the 14-day postoperative period, reflecting treatment-related toxicity, was also more pronounced in the HIPEC groups. However, when looking at the PCI score at autopsy, the extent of disease was significantly lower when rats were treated with CRS and HIPEC as compared to CRS alone. Furthermore, within each treatment group, PCI was significantly lower at autopsy as compared to day of treatment when rats received CRS and HIPEC. Regarding weight loss, extent of disease and survival, there was no difference between rats treated with BSA-based or concentration-based HIPEC. We concluded that clinical parameters, including weight loss and extent of disease are not predictive for tumor response.

In conclusion, this study clearly demonstrates that there is no difference in PA between BSA-based and concentration-based HIPEC. Moreover, the PA does not provide any information about the true pharmacodynamic efficacy of the drug to treat the tumor nodule in the peritoneal cavity. We provide proof of principle that a higher IP concentration of the chemotherapeutic drug in the concentration-based HIPEC results in a higher concentration of the drug in the tumor nodule at the end of the 30-minute HIPEC procedure. We emphasize the importance of considering the tumor nodule as the pharmacologic endpoint rather than the PA of the drug. We considered apoptosis as a potential pharmacodynamic endpoint in order to try to correlate pharmacokinetic and pharmacodynamic parameters in determining tumor response. The next step is the translation of the optimization and standardization of the HIPEC dosing regimen in a clinical setting taking into account the individual drug sensitivity in the treatment of PSM.

## MATERIALS AND METHODS

### Ethics statement

Research has been conducted in accordance with the ethical standards, conforming to the EU Directive 2010/63/EU for animal experiments and was approved by the local Ethical Committee for Animal Experiments at Hasselt University, Belgium (protocol number: 201734).

### Safety considerations

When working with chemotherapeutic agents, standard safety precautions were applied. These include wearing personal protective equipment (eye protection, protective gloves, mouth mask and protective clothing) and using standardized handling procedures, including the use of BD PhaSeal™ closed system transfer devices (Dublin, Ireland), to minimize chemotherapy associated exposure risks. All chemotherapy associated materials and animal samples were disposed in WIVA medical waste containers.

### Cancer cell line

#### Cell culture

The syngeneic rat colorectal carcinoma CC-531 cell line was kindly provided by the Research Laboratorium Surgery of the Radboud University Medical Center in the Netherlands. Cells were cultured in RPMI 1640 medium (BE12-115F/U1, BioWhittaker^®^, Lonza, Verviers, Belgium), supplemented with 10% fetal bovine serum (FBS, Gibco^®^, Life Technologies, Paisley, UK) and 1% penicillin-streptomycin (Sigma-Aldrich, St. Louis, Missouri, USA), under standardized incubator conditions, 37°C and 5% CO2. Cell suspensions were prepared after enzymatic detachment with trypsin-EDTA solution (Sigma-Aldrich, St. Louis, Missouri, USA). Subsequently, the cell suspension was centrifuged at 1000 rpm for 2 minutes and resuspended in supplemented RPMI 1640 medium to reach the required concentration. Cell suspensions for animal injection were resuspended in 2 mL phosphate buffer saline (PBS) solution (BE17-516F, BioWhittaker^®^, Lonza, Verviers, Belgium) at a concentration of 10^6^ cells/mL.

#### Oxaliplatin *in vitro* cytotoxicity

Viability of the CC-531 cell line after oxaliplatin (5 mg/ml Eloxatin^®^, Sanofi, Diegem, Belgium) treatment was evaluated *in vitro* by the colorimetric MTT assay. CC-531 cells were seeded in 96-well plates (Cellstar^®^, Greiner Bio-One GmbH, Frickenhausen, Germany) at a density of 8000 cells/100 μL/well and incubated at 37°C, 5% CO2 for 24h. Medium was replaced by 200 μL oxaliplatin, 10-fold diluted in medium to reach concentrations of 20 μg/mL, 30 μg/mL, 50 μg/mL and 75 μg/mL; corresponding to the following dosages of 40 mg/m^2^, 60 mg/m^2^, 100 mg/m^2^ and 150 mg/m^2^, in 2 L/m^2^. Next, the plates were incubated at standardized incubator conditions for 30 minutes. Subsequently, the chemotherapy solution was replaced by 200 μL medium and incubated at standardized incubator conditions for 24 hours. Afterwards, 10 μL MTT solution (Life Technologies, Eugene, Oregon, USA) was added and incubated at standardized incubator conditions for 4h, protected from light. The medium was removed and the formazan crystals were dissolved in 100 μL sodium dodecyl sulfate (SDS, Life Technologies, Eugene, Oregon, USA). Eight wells per concentration were used and all experiments were performed in triplicates. The absorbance of samples was measured at 570 nm with a FLUOstar^®^ Omega microplate reader (BMG Labtech, Offenburg, Germany). Cell viability of oxaliplatin treated cells compared to control cells, treated with medium without the chemotherapeutic agent, was expressed in percentages.

### Animals and housing

Ten weeks old male WAG/Rij rats with a median weight of 236.6 (219.8 – 248.10) g were obtained from Charles River Laboratories (Calco, Italy). Rats were accustomed to laboratory conditions for 1 week before experimental use and housed under clean, nonsterile standardized conditions (temperature 22°C, 12h light/12h dark) in Eurostandard type IV open-top cages (three rats per cage) with autoclaved sawdust bedding and cage enrichment. The animals were allowed free access to food (2018 Teklad global 18% protein rodent diet, Envigo, Madison, Wisconsin, USA) and acidified water.

### Anesthesia

All experiments; i.e. induction of PSM, CRS and HIPEC were performed under anesthesia using a mixture of isoflurane (IsoFlo^®^, Zoetis, Louvain-la-Neuve, Belgium), 1 – 2% volume supplemented with oxygen. Anesthesia was induced in an induction chamber (3.5% volume supplemented with oxygen) and continued by a face mask (1 – 2% volume supplemented with oxygen). Level of anesthesia was assessed by evaluation of motoric response to a toe pinch. During all experiments, rats were put into a supine position onto a heating pad.

### Induction of peritoneal surface malignancy

PSM was induced by IP injection of 2 mL cell suspension in PBS of the syngeneic colorectal carcinoma rat cell line CC-531 at a concentration of 10^6^ cells/mL. The injection was performed under induction of anesthesia.

### Study design

#### Maximum tolerated dose

The MTD of oxaliplatin was evaluated in rats treated with CRS and HIPEC. MTD was defined as the highest non-lethal dose of oxaliplatin at which the humane endpoints were not reached. These included extensive weight loss of more than 20% compared to the body weight measured at day of operation, during three consecutive days within two weeks. Eight days post IP injection of the CC-531 cell line, 12 rats were randomized to CRS and HIPEC at the following increasing oxaliplatin concentrations: 40, 60, 100 and 150 mg/m^2^ in 2 L/m^2^ 0.9% (w/v) NaCl carrier solution.

#### Body surface area-based versus concentration-based HIPEC

Eight days after IP injection of the CC-531 cell line, 63 rats were randomized into three groups:

- CRS group (n=21): exploration and CRS alone.
- HIPEC-BSA group (n=21): CRS followed by HIPEC using the BSA-based dosing method, total dose of 150 mg/m^2^ oxaliplatin. BSA of the animals was calculated by means of the Du Bois, Du Bois formula: BSA = 0.007184 x Weight^0.425^ x Height^0.725^ [31].
- HIPEC-CONC group (n=21): CRS followed by HIPEC using the concentration-based dosing method, 150 mg/m^2^ oxaliplatin in 2 L/m^2^ carrier solution resulting in a fixed concentration of 75 mg/L oxaliplatin.

Primary endpoint was a difference in PA. Secondary endpoints were efficacy and survival.

### Cytoreductive surgery

Eight days after PSM induction, CRS was performed under general anesthesia. Thirty minutes prior to surgery, rats were given a subcutaneous injection of buprenorphine (Temgesic^®^, RB Pharmaceuticals Limited, Berkshire, UK) 0.1 mg/kg/day, and thereafter once daily until the third postoperative day. First, a midline laparotomy was performed and the abdomen was carefully inspected for tumor growth at 12 different sites (Table 1). Tumor deposits at each site were scored semiquantitatively: 0 for no macroscopic tumor, 1 for limited tumor growth (1–2 mm Ø), 2 for moderate tumor growth (2–4 mm Ø), or 3 for extensive tumor growth (>4 mm Ø). The sum of scores from all sites represented the modified PCI, based on the PCI score developed by Sugarbaker to evaluate disease burden [16, 32]. Subsequently, surgery involved standard omentectomy, splenectomy and resection of the gonadal fat pads using an electrocoagulation device. When macroscopic tumor nodules were found in the abdominal fat pads, the latter were removed aiming at complete macroscopic resection. No bowel resections or anastomoses were made. Residual tumor load was scored using the R1–R2a–R2b classification. Absence of residual tumor was scored as R1, a residual tumor of 2.5 mm or less was scored as R2a, and a tumor larger than 2.5 mm as R2b.

### Hyperthermic intraperitoneal perioperative chemotherapy

HIPEC in rats was performed mimicking the open coliseum technique used in current clinical practice. No warming mattress was used during HIPEC, to avoid systemic hyperthermia. The skin edges of the abdomen were raised and attached to a metal frame using sutures. Two sterile Intrafix^®^ SafeSet infusion drains (B. Braun, Melsungen, Germany) were placed in the abdomen and secured on the frame using sutures. These infusion drains were attached to BD Insyte™ IV catheter 22-gauge needle covers (381223, Sandy, Utah, USA). The infusion drains were connected to a closed perfusion system containing a total volume of 250 mL 0.9% (w/v) NaCl (Baxter, Lessines, Belgium) carrier solution. The inflow and outflow drains were placed at opposite sides of the abdomen to achieve a uniform heat distribution and to avoid microcirculation of the perfusate. The peritoneal perfusate was warmed in a glass bottle using a thermostatically regulated water bath (58°C), aiming at a perfusate temperature of 41.5°C. The perfusate was infused into the abdomen at 10 mL/min (33 rpm) during 30 minutes, using a 120U/DM2 peristaltic pump (010.6141.M20, Watson-Marlow, Zwijnaarde, Belgium). When stable perfusion conditions were achieved and stable perfusate temperature was reached, oxaliplatin was diluted in the circulating carrier solution to the correct concentration, either BSA-based or concentration-based. An extractor hood was placed above the animal during the perfusion procedure to evacuate drug vapor and protect the researchers present. During HIPEC, rectal and intra-abdominal temperatures were monitored every 5 minutes using a rodent thermometer (BIO-TK8851, Bioseb, Vitrolles, France). At each 10-minute time interval, blood (400 μL) and peritoneal fluid (200 μL) were sampled for Pt quantification using a previously validated ICP-MS method (see section 4.11) [33]. Blood was sampled by means of jugular vein catherization using the catheter rat jugular vein, PU 3Fr 12 cm, collar at 3.8 cm (Instech Laboratories, Pennsylvania, USA); allowing repeated sampling. Peritoneal fluid was sampled directly in the abdomen of the rat. Omental tumor nodules were sampled at the end of the 30-minute HIPEC procedure for both ICP-MS analysis and IHC apoptosis quantification (see sections 4.11 and 4.12). HIPEC protocol was standardized to take the first sample 2 minutes after administration of oxaliplatin (start 30-minute perfusion clock) to allow homogenization of the chemotherapy solution. After 30 minutes, the perfusate was evacuated by suction, the infusion drains were discarded and the abdominal wall was closed in two layers using continuous 4/0 polyglactin 910 sutures (Vicryl Rapide™, Ethicon, Somerville, New Jersey, USA) for the muscular layer and the skin layer. Additional interrupted sutures were placed between the continuous sutures for the skin layer. The animals were given 5 mL 0.9% (w/v) NaCl subcutaneously for rehydration.

### Follow-up

Rats, treated with CRS and HIPEC (to determine the MTD of oxaliplatin), were observed and weighted daily for 14 days after surgery and thereafter euthanized. Rats of the CRS, HIPEC-BSA and HIPEC-CONC group were observed and weighted daily for 14 days after surgery and two times per week thereafter. To reflect the toxicity of the treatment, body weight was expressed as relative body weight compared with the body weight on the day of surgery. Food was placed on the bottom of the cage and water was supplied using a drinking bottle with a long spout. When the humane endpoints were reached, animals were euthanized by IP injection of 200 mL/kg pentobarbital (Val d’hony Verdifarm, Beringen-Paal, Belgium) under induction anesthesia. Humane endpoints included extensive weight loss of more than 20% (MTD group) or 25% regarding the body weight measured at day of operation, during 3 consecutive days within two weeks. Other humane endpoints were presentation of abnormal behavior and lack of grooming during 3 days after termination of analgesia. Remaining animals were euthanized 140 days post-surgery, when the study was terminated. When possible, animals were subjected to autopsy and tumor load was evaluated using the modified PCI score as described in section 4.8.

### Inductively coupled plasma mass spectrometry

Pt quantification was performed using a previously validated ICP-MS assay [33]. In summary, after the CRS and HIPEC procedure, blood samples were centrifuged for 10 minutes at 1500 rpm at room temperature. The resulting plasma, peritoneal fluid and tumor nodules were stored at -80°C until day of ICP-MS analysis. Tumor nodules were digested in 25% hydrogen peroxide (Merck KGaA, Darmstadt, Germany) and in nitric acid (J.T. Baker, Avantor Performance Materials, Pennsylvania, United States) using the High Performance Microwave Digestion System from Milestone, Ethos Up. Digestion was performed according to the following schedule: 0-15 minutes, 1800W, ↑200°C; 15-30 minutes, constant temperature at 200°C; followed by a cool down period. Afterwards the mixture was 10-fold diluted before ICP-MS analysis. Sample preparation of the plasma and peritoneal fluid samples involved a simple 1000-fold dilution in 0.5% nitric acid. The ICP-MS system consisted of a Perkin Elmer NexION 350S system equipped with the Syngistix software version 1.1. and an ESI Prep-Fast delivery system controlled by the ESI SC software version 2.9.0.202. The analytes, isotopes of Pt and the internal standard terbium (Tb) were monitored at m/z Pt 194, Pt 195 and Tb 159. Pt concentration in the tumor nodules was expressed as ng/mg wet tissue. Pt concentration in plasma and peritoneal fluid samples was expressed as μg/mL.

### Immunohistochemistry for activated caspase-3

Apoptosis in the center and outer layer of each tumor nodule (sampled from the greater omentum) was evaluated by means of IHC staining for activated caspase-3. Ten μm frozen sections were stored at -80°C until day of analysis. Sections were washed for 5 minutes with aqua destillata followed by cell permeabilization for 5 minutes using PBS-0.03% Triton X-100 (Fluka, Sigma-Aldrich Chemie, Steinheim, Germany). Non-specific interactions were minimized through blocking by incubation in 0.3% hydrogen peroxide (Sigma-Aldrich Chemie, Steinheim, Germany) in methanol (VWR Chemicals, Fontenay-sous-Bois, France) for 20 minutes, and subsequently incubated with 20% pre-immune goat serum for 45 minutes. Thereafter, sections were incubated overnight with the primary antibody rabbit active caspase-3 (1/20 dilution, 3015-100, Gentaur, Kampenhout, Belgium). Next, sections were incubated with the secondary antibody, goat anti-rabbit biotin (E043201-8, Agilent Technologies, Diegem, Belgium) for 45 minutes followed by a 30-minute incubation with Streptavidin-HRP (1/100 dilution, Dako, Glostrup, Denmark) and 8 minutes incubation with the TSA™ Cyanine 3 kit (NEL704A001KT, Perkin-Elmer, Nossegem, Belgium). Cell nucleus was stained by means of DAPI staining (1/1000 dilution in PBS) for 10 minutes. Every step was interspersed with 3 times 5-minute wash steps in PBS. For negative controls, the primary antibody was omitted. Microscopy was performed using a ELYRA PS.1 epi-fluorescence microscope (Carl Zeiss, Jena, Germany). Wide-field images of whole tumor cross sections were obtained by means of tile scans using a Plan-Apochromat 20x/NA0.8 Ph2 objective. DAPI and Cy3 were sequentially excited with lasers at 405 nm and 561 nm respectively. The emission light of DAPI was collected at 420 – 480 nm and the Cy3 emission light was collected at 570 – 620 nm. The acquired image resolution for each tile was 1280×1280 pixels^2^ at a pixel size of 203 nm. For regions with very high cell density, additional images were acquired with structured illumination microscopy using a Plan-Apochromat 63x/NA1.40 Oil DIC (Carl Zeiss, Jena, Germany) objective utilizing the same optical filters as described above, resulting in images with 1280×1280 pixels^2^ at a pixel size of 64 nm. For each structured illumination image, 25 raw images (combination of 5 angles and 5 translations of the diffraction grating) were recorded and subsequently reconstructed into a final high resolution image using automatic 2D processing in ZEN software (version 2011, Carl Zeiss, Jena, Germany). Cells positive for activated caspase-3 were counted manually in two sections for each tumor nodule (center and outer layer) and expressed as number of cells/mm^2^.

### Statistical analysis

Statistical analysis was performed using the SPSS^®^ version 25.0 (IBM Corp., Armonk, NY, USA). The primary objective of this preclinical study was a difference in PA. Sample size was calculated using the statistical program G*power version 3.0.10 (Heinrich-Heine-Universität Dusseldorf). A pilot study was conducted; rats were treated with CRS and oxaliplatin-based HIPEC, BSA-based (n=5) or concentration-based (n=5). The PA was calculated for each of these rats. As these data were normally distributed, sample size was calculated by means of a t-test using a power of 0.90, alpha of 0.05 and a calculated effect size of 1.04. Equal distribution was tested with the Shapiro-Wilk test. Depending on normality, Student's t-tests or Mann-Whitney U tests were used to compare continuous data between two treatment groups. One-way ANOVA with post hoc Tukey or Bonferroni and Kruskal-Wallis test were used for continuous data between three groups. The Wilcoxon singed-rank test was used to compare two related samples within each treatment group. Spearman's rank correlation was used to test for possible correlations. Survival analysis was performed using Kaplan-Meier curves and compared by means of log-rank test. Cox survival regression analysis was used to correct for confounding factors. A *p* value <0.05 was considered statistically significant.

## Abbreviations

AUC: area-under-the-curve
BSA: body surface area
c.i.: confidence interval
CRS: cytoreductive surgery
HIPEC: hyperthermic intraperitoneal perioperative chemotherapy
ICP-MS: inductively coupled plasma mass spectrometry
IHC: immunohistochemistry
IP: intraperitoneal
MTD: maximum tolerated dose
MTT: 3-[4,5-dimethylthiazol-2-yl]-2,5-diphenyltetrazolium bromide
PA: pharmacologic advantage
PCI: peritoneal cancer index
PSM: peritoneal surface malignancy
Pt: platinum
Tb: terbium.
